# Understanding the Thermodynamics of Magnesium Binding to RNA Structural Motifs

**DOI:** 10.3390/life14060765

**Published:** 2024-06-16

**Authors:** J. A. Cowan

**Affiliations:** Department of Chemistry and Biochemistry, The Ohio State University, 100 West 18th Avenue, Columbus, OH 43210, USA; cowan.2@osu.edu

**Keywords:** divalent magnesium ion, RNA, structural motifs, thermodynamics, binding affinity, Mg^2+^

## Abstract

Divalent magnesium ions (Mg^2+^) serve a vital role in defining the structural and catalytic chemistry of a wide array of RNA molecules. The body of structural information on RNA motifs continues to expand and, in turn, the functional importance of Mg^2+^ is revealed. A combination of prior work on the structural characterization of magnesium binding ligands with inner- and outer-sphere coordination modes, with recorded experimental binding energies for inner- and outer-sphere contacts, demonstrates the relative affinity and thermodynamic hierarchy for these sites. In turn, these can be correlated with cellular concentrations of free available magnesium ions, allowing the prioritization of populating important functional sites and a correlation with physiological function. This paper summarizes some of the key results of that analysis and provides predictive rules for the affinity and role of newly identified Mg binding sites on complex RNA structures. The influence of crystal packing on magnesium binding to RNA motifs, relative to their solution form, is addressed and caveats made.

## 1. Introduction

The prevalence and important functional role of divalent magnesium binding to nucleic acids and nucleic acid polymers (DNA and RNA) is well recognized [1,2,3,4,5]. RNA, with a highly complex tertiary structure and a more diverse degree of functional roles (informational, catalytic, regulatory) [5], is by far the more interesting case for consideration of metallonucleic acid coordination chemistry. While the chemical and structural ramifications of metal binding to both simple and complex nucleic acids has been the focus of extensive study with regard to natural functional roles [1,2,3,4,5,6,7,8,9,10,11,12,13,14,15,16], such as the cases of Na^+^, K^+^, Mg^2+^, and Ca^2+^ [16], and the chemistries of heavy and softer elements related to toxicity or medicinal roles, such as Pt^2+/4+^, Cd^2+^, Tl^+^, etc. [17], it is the divalent magnesium ion that controls much of the structural and functional chemistry of natural nucleic acids in their physiological setting. In this paper, the focus lies on Mg^2+^ interactions with RNA. The natural selection of divalent magnesium reflects the relatively high physiological concentration of a free and available cation (~0.5 mM), and its high Lewis acidity that results from the divalent charge and smaller ionic radius [16,17]. These two factors provide a stronger binding affinity and more effective catalysis, when required, and emphasize the importance of understanding the coordination chemistry of magnesium binding to structurally and functionally complex RNA motifs. Herein, the results of an analysis of magnesium binding sites on previously reported RNA binding motifs [5] are presented with identified inner- and outer-sphere coordination modes, allowing a detailed characterization of the relative affinity of RNA magnesium binding domains and a correlation with physiological function. This paper summarizes key results of that analysis and provides predictive rules for the affinity and role of newly identified Mg^2+^ binding sites on complex RNA structures.

## 2. Materials and Methods

The analyses in this paper build on a summary of thousands of structurally characterized magnesium binding sites identified in RNA crystal structures as presented by Minor and coworkers [5]. Most of these structures incorporated proteins and peptides that were important for the definition of overall structure, and therefore metal binding motifs. Inasmuch as these did not represent “clean” metal binding motifs, the majority of these structures were omitted for the purpose of this analysis. Approximately 400 sites remained and are tabulated in a detailed fashion in the Supplementary Materials file (Table S1). The data documented in the analysis by Minor and coworkers [5] was downloaded and worked-up in excel mode, and the data then parsed according to inner-sphere and outer-sphere coordination modes, and by reference to the specific binding ligands. Categorization of sites by distinct inner-sphere classes was also considered [5]. 

**Figure 1:**
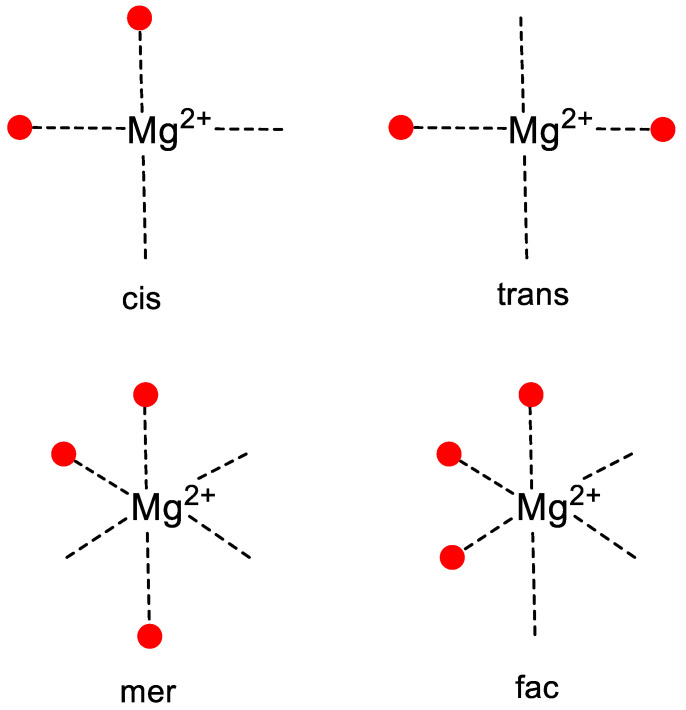
Common coordination motifs for divalent magnesium ion, where the red dots represent bound ligand atoms or ions and the relative positioning of the ligands around the coordination sphere represent the binding geometries conveyed by the terms cis or trans for a planar arrangement of RNA contacts, and mer or fac for an array based on an octahedral geometry.

Common coordination motifs for divalent magnesium are illustrated in Figure 1 and defined in Table 1 and Table 2. Thermodynamic binding parameters were evaluated on the basis of a prior delineation of inner-sphere and outer-sphere binding energies (Table 1) [18] that allowed for an ordering of the thousands of bound Mg^2+^ ions based on relative total binding energy. While other computational estimates of inner- and outer-sphere binding energies have been reported [4,12], selection of parameters from such studies would not impact the conclusions made in this work, since each inner-sphere binding energy always substantially outweighs that from an outer-sphere contact. The resulting data file is found in Supplementary Information (Table S1), and a summary table for select examples to be discussed in this paper is presented in Table 3.

## 3. Results

The structural information provided by Minor and coworkers [5] was parsed according to the nature of the bonding mechanism (inner- versus outer-sphere) for a divalent magnesium ion bound to a variety of functional RNA moieties (including riboswitches, pseudoknots, ribosomal, ribozymes, etc.). Inner-sphere binding by phosphate oxygen, base heteroatoms, or ancillary bound ligand atoms was distinguished, as was outer-sphere hydrogen bonding from Mg^2+^-bound water to backbone phosphates, ribose hydroxyl, or other base or ligand heteroatoms. The binding energy for each inner-sphere contact was taken as −3.3 kcal mol^−1^ (Table 1). A general value was required since no detailed information is available for discrete classes of phosphate or heteroatom coordination to Mg^2+^; however, this seems acceptable in light of the marked difference between the binding energies for inner- and outer-sphere contacts (Table 1). The sizable difference in inner- and outer-sphere binding energies accommodates distinctions in the ligands bound through inner- or outer-sphere mechanisms.

From the data available from the analysis of Minor et al. [5], approximately 15,535 RNA motifs were downloaded and screened. The vast majority of the structures that laid the foundation for this published database were of protein-complexed RNAs. Since the role and energetic contribution of the bound protein to stabilizing each magnesium complexed RNA motif is unknown, only the protein-free RNA moieties (or cases where protein binding was distinct from bound Mg^2+^) were considered in the analysis presented herein. This provided approximately 400 sites for consideration and subsequent thermodynamic analysis. The binding energies for each site were calculated by summing the total number of inner- and outer-sphere contributions, using the experimental thermodynamic data summarized in Table 1. The overall summary of the magnesium binding sites, bound ligands, and mode of binding, as well as the calculated binding free energies, is provided in the Supplementary Information file (Table S1). Key data that pertains to the analysis and discussion of the specific examples analyzed in this manuscript is presented in Table 3.

## 4. Discussion

### 4.1. Significance of Binding Affinity

**Figure 2:**
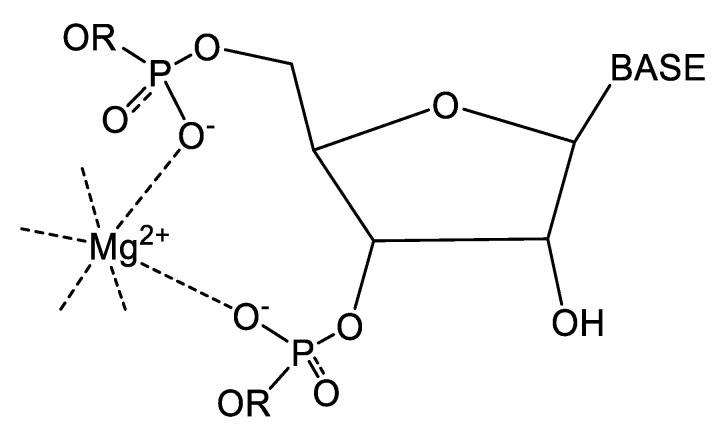

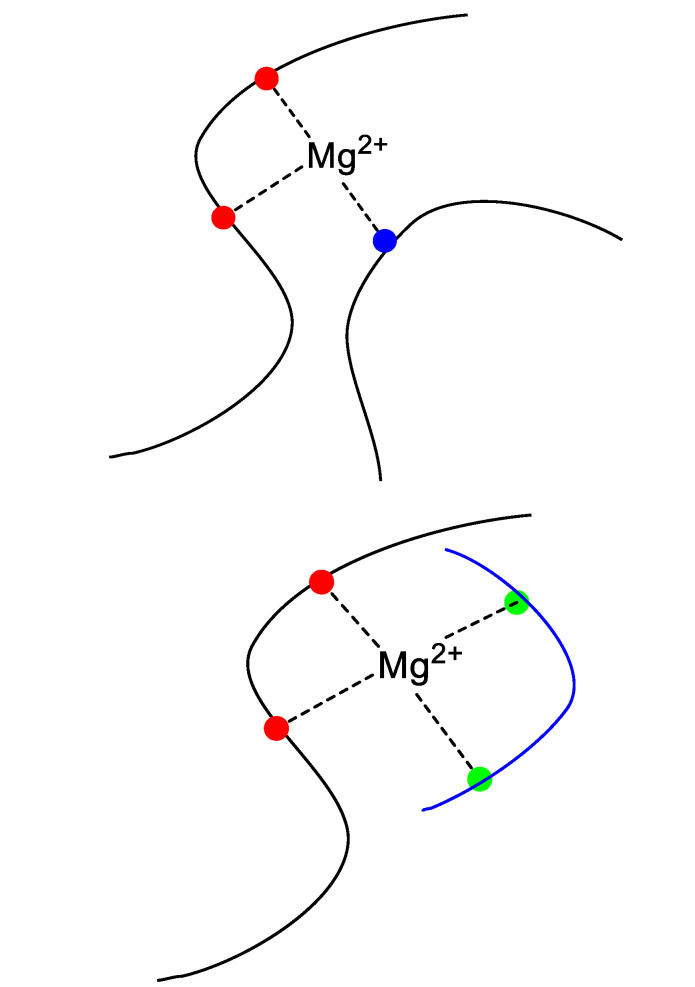
(TOP) A representation of the 10-membered-ring magnesium binding motif (reflecting the 10 atomic centers that make up the binding structure) [5]. The divalent magnesium ion is coordinated by two adjacent phosphates in the sugar–phosphate backbone in a cis geometry (Figure 1). While the magnesium- and Y-clamp Mg^2+^ binding motifs [5] may also exhibit trans-, mer-, or fac-coordination geometries (Figure 1), these also incorporate a cis motif within the “10-membered” ring component. (MIDDLE) An illustration of the relationship of the core 10-membered motif to the Y-clamp binding geometry [5], where a bridging magnesium ion crosslinks two distinct RNA strands (herein depicted schematically as lines rather than explicitly showing the structural details of the sugar–phosphate backbone); alternatively, it binds a phosphate further along a strand, and in either case adopts a “mer” or T-shaped geometry (Figure 1). The two bound phosphates from the 10-membered ring are represented by red dots and the phosphate from the additional phosphate moiety is depicted by the blue dot. (BOTTOM) An illustration of the relationship of the core 10-membered motif (Figure 1) to the magnesium-clamp binding geometry, representing a case where a bridging magnesium ion crosslinks two distinct RNA strands (colored black and blue), each adopting a 10-membered motif to the Mg^2+^ ion (Figure 1). The two bound phosphates from the 10-membered ring are represented by red dots on one strand and green dots for the other.

The data summarized in the Supplementary Materials file (Table S1), and the selected examples taken for consideration herein (Table 3), all clearly demonstrate that the highest affinity binding sites stem from direct inner-sphere coordination by a divalent magnesium ion. Most commonly, this involves direct phosphate–oxygen coordination, and infrequently, ribose hydroxyl, a base heteroatom, or exogenous ligand binding. These binding sites also encompasses the metal binding motifs previously characterized as the magnesium clamp [19], the 10-membered ring, and the Y clamp (Figure 1 and Figure 2, and Table 2) [5]. The most fundamental of these is the 10-membered ring, which is the equivalent in RNA chemistry of the “cis” chelate identified in classical coordination chemistry [20]. The distinctions between the magnesium-clamp and Y-clamp motifs reflect additional bound phosphates that may lie close to the nucleotides that form the 10-membered ring, or can be many base units along the chain, or even from a distinct RNA chain. The structural roles that each scenario represents would be either to induce a bent structural motif or to draw two distinct chains or a distant part of a chain closer together (Figure 2). Given that such bound Mg^2+^ ions not only serve to overcome electrostatic charge repulsion, but also to accommodate the steric strains that can accompany such structural changes, the requirement for enhanced binding affinity is clear. The highest affinity Mg^2+^ sites are therefore directly associated with these types of structural motifs (kink turns, pseudo-knots, etc.) and define folding patterns in ribosomal RNAs, transfer RNAs, riboswitches, and ribozymes. 

As one descends the order of highest affinity sites (Table 3 and the Supplementary Materials file under Table S1), one moves from a preponderance of inner-sphere to outer-sphere coordination modes (Table S1, columns L, M, and N, and Figure S1; all found in the Supplementary Materials file). The former most often reflect crosslinking interactions that bring together portions of the RNA backbone and define tertiary structure. The latter serve distinct roles and are typical of charge-neutralization sites, where divalent magnesium is serving to balance charge on the polyphosphate backbone and where hydrogen-bonding to magnesium-bound water molecules provides a framework for multiple outer-sphere contacts. Lacking a need to stabilize structure, such sites consequently exhibit weaker binding. Moreover, not all of these sites need be populated to effect charge neutralization, and so weaker binding is again excused. At the end of the spectrum, where ∆G ≥ −9 kcal/mol, the coordination mode encompasses both minimal inner-sphere and compensatory outer-sphere modes.

### 4.2. Binding Affinity versus Cellular Concentration of Mg^2+^(aq)

While concentrations of magnesium ions may vary according to the organ or cell type involved, the concentration of free available Mg^2+^(aq), which is the level that all bound ions must be in dynamic equilibrium with, is around 0.2–0.5 mM [21]. With that in mind, the higher affinity sites, with a free energy of binding (∆G_b_) ~ −16.5 kcal mole^−1^, are all equilibrated with the maximal available cellular concentration of magnesium ions. That is, Mg^2+^ that is not already complexed with other biological ligands. Such sites are evolutionarily designed to complex an essential and functional divalent ion for structural or catalytic roles and are optimized for binding at the physiological concentrations of available Mg^2+^(aq). The highest affinity sites are also identified with stabilizing structural motifs with significant steric strain and/or loss of molecular entropy. In the case of single-strand domains, such sites may also substitute for the loss of base-pairing energy when secondary structures adapt to a functional need to bind Mg^2+^. 

Moving down the binding spectrum, with ∆G_b_ ~ −10 kcal mole^−1^, there is found increasing levels of outer-sphere coordination that corresponds to charge neutralization. In fact, a common role for Mg^2+^ in both ribonucleic acid and deoxyribonucleic acid polymers is in simple charge neutralization of the polyphosphate backbone, with outer-sphere binding to multiple points of contact providing higher overall affinity to a polynucleotide strand, relative to direct coordination to a specific phosphodiester functional group.

### 4.3. Illustrative Examples of Magnesium Binding Motifs

**Figure 3:**
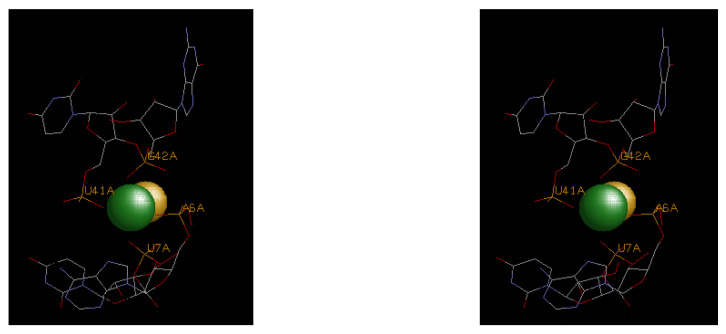
A stereo view of the divalent magnesium ion (green) coordinated in the fluoride riboswitch (4ENC). Principal coordination is by the phosphates at bases A6, U7, U41, and G42. The latter are represented in stick format with normal CPK color representations. Labeling is provided for the key base positions. Both of the bidentate phosphate chelate moieties (A6/U7 and U41/G42) represent 10-membered-ring motifs and together are arranged in a planar fashion. The fluoride ion (yellow) is coordinated in an axial manner.

**Figure 4:**
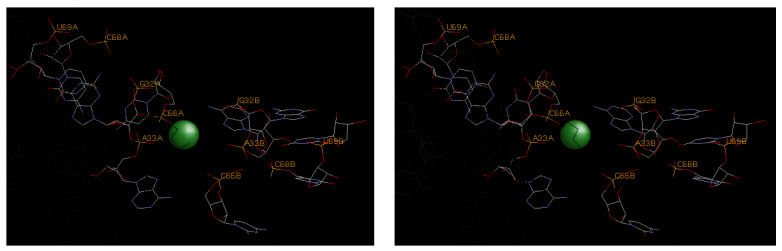
A stereo view of the divalent magnesium ion (green) coordinated in the glycine riboswitch (3OXM). Principal coordination is by the phosphate at base A33 and ribose oxide at G32 associated with strand B, as well as the phosphate from base C66 on a distinct strand A. This would correspond to a type of Y-clamp motif, where the ribose hydroxyl coordinates in place of the backbone phosphate. Additional stabilization also derives from outer-sphere contacts from heteroatoms on bases U67, C68, and U69 on strand A. These base positions are represented in stick format with normal CPK color representations and labeling is again provided for the key base positions.

**Figure 5:**
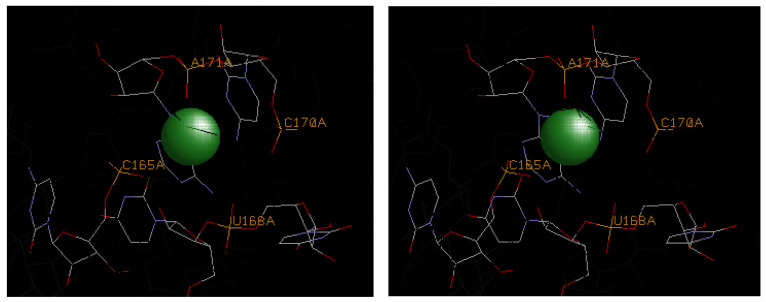
A stereo view of the divalent magnesium ion (green) coordinated in the illustrated ribozyme motif (1HR2). A single inner-sphere contact is made to the phosphate at base position A171. Multiple outer-sphere hydrogen-bonding contacts are made to phosphates at base positions C165, U168, and C170, and to a ribose hydroxyl at U167 and base heteroatoms at U167 and A171. Key base positions are highlighted in stick format with normal CPK color representations and labeling is provided.

It was earlier noted that the magnesium clamp, the 10-membered ring, and the Y-clamp (Figure 2 and Table 2) represent the most common motifs for magnesium binding; however, other less common, but high-affinity sites are also identified in Table 3. These sites exhibit binding to base heteroatoms and more extensive hydrogen bonding that reflect unique binding scenarios with enhanced steric strain and electrostatic repulsion from closely associated backbone phosphates. Examples of each of these motifs are shown in Figure 3, Figure 4 and Figure 5.

The fluoride binding riboswitch in Figure 3 illustrates the classical 10-membered-ring motif, with two pairs of adjacent backbone phosphates arranged in a planar coordination arrangement that leaves an axial site for the bound fluoride ions. 

Figure 4 illustrates a Mg^2+^ site on a glycine riboswitch. This structure highlights two nuances of magnesium coordination motifs and one caveat. First, the 10-membered-ring coordination motif can incorporate a ribose oxygen ligand in place of the backbone phosphate, but the energy to ionize is offset by the second factor, namely outer-sphere coordination to base heteroatoms. The caveat stems from the involvement of two symmetry-related RNA chains. Such symmetry reflects crystal packing forces that are almost certainly not involved in the solution chemistry of this RNA moiety and one might therefore conclude that this bound magnesium is of dubious functional relevance.

Finally, Figure 5 shows a divalent magnesium site in a structure determined for a ribozyme that is primarily stabilized by multiple outer-sphere H-bonding interactions; thereby maintaining a relatively high binding affinity (∆G ~ −10.5 kcal/mol, Table 3) in a localized coordination setting encompassing nucleotide positions 165–171. Such a site normally serves to provide electrostatic balance to a pocket of negatively charged phosphates.

### 4.4. Mg^2+^ Binding Energetics

Such structural data suggests that the primary motivation of critical cellular activities is to create a coordination environment for divalent magnesium that is capable of binding and sustaining the key catalytic and/or structural requirements of a functional motif under the conditions of available free Mg^2+^(aq). The data in Table 1 and Table 3 indicate that this is often promoted by virtue of direct phosphate coordination, but can also be mediated by other base heteroatoms or outer-sphere contacts to phosphates, ribose hydroxyls, or heteroatoms. As such, the functional relevance of established magnesium binding motifs must be viewed through the lens of binding thermodynamics rather than structural terms. A variety of structural motifs could be used to promote magnesium coordination (∆G_coord_) depending on the outcome of Equation (1), which delineates net binding energy (∆G_b_) relative to energy loss from steric constraints (∆G_steric_) that reflects the strain imposed by the required structural motif for functional utility, and the electrostatic repulsion from backbone phosphates (∆G_elec_). These represent the primary energy contributions that dictate the overall binding energy (∆G_b_), which is represented by the sum of a favorable coordination term (∆G_cord_) and unfavorable steric energy terms (∆G_steric_ and ∆G_elec_). Neither of these latter individual terms are directly measurable. An additional term for electrostatic repulsion of adjacent bound Mg^2+^ could also be included, but contiguous cations are rarely found in a specific RNA molecule and this term can be reasonably omitted. While averaged crystallographic data suggest the presence of multiple neighboring cations, most of the Mg(II) sites are populated at fractional percentages; that is, in most cases the unit cells would not contain a bound ion. Moreover, the vast majority of the sites will not be populated at physiological Mg(II) concentrations, are not functionally relevant, and will not coexist with neighboring sites.

∆G_b_ = ∆G_coord_ − ∆G_steric_ − ∆G_elec_ (1)

### 4.5. Comparison with Earlier Structural and Thermodynamic Studies of Divalent Magnesium Complexes with RNA

Early studies by the author established the importance of the outer-coordination hydration sphere in promoting magnesium ion binding to polynucleotides, and in mediating enzymatic activity toward nucleic acid substrates [6,7,8,16,21,22,23,24]. The use of cobalt hexaammine [Co(NH_3_)_6_^3+^] as a probe of the binding and functional activity of magnesium-derived outer-sphere contacts was established and has been further utilized in both structural and functional studies [10,11,18,22]. Tinoco and coworkers demonstrated Co(NH_3_)_6_^3+^ as a probe of divalent metal ion stabilization of RNA strands and structural motifs, including pseudoknots and ribozymes, utilizing the slow exchangeability of bound ammonia protons in nuclear magnetic resonance (NMR) experiments to investigate potential Mg(H_2_O)_6_^2+^ binding sites [10,11]. It is clear from the truncated Mg^2+^ data base presented in Table S1 in the Supplementary Materials file that almost all of the high-affinity divalent metal binding sites are defined by at least two inner-sphere contacts that are majorly from phosphates. Nevertheless, outer-sphere contacts do make a significant contribution to the stabilization of a variety of functional RNA motifs. The lower binding energy for such sites is most likely a simple reflection of the role of other stabilizing interactions from internal RNA H-bonding networks, with the charged cation serving a charge stabilization role rather than promoting cross-linking interactions within the RNA motif. 

An example of such a role is also demonstrated by the formation of metal ion binding pockets around RNA kissing loops, where two hairpin loops are cross-connected by base pairing interactions [15]. In such cases the resulting electrostatic backbone repulsion is stabilized by outer-sphere Mg(H_2_O)_6_^2+^ binding sites. In fact, the binding affinity of inner- and outer-sphere Mg^2+^ is also critically dependent on the location of the bound ion, either exterior or inner surface, which most likely reflects the negative charge density and the availability of inner- and outer-sphere ligand contacts [4,12].

Transient metal binding interactions in controlling dynamic structural changes in RNA motifs have also been well documented [1]. In that regard, Roy et al. have described experimental and computational studies of how Mg^2+^ promotes preorganization of an RNA triplex in the SAM-II riboswitch [2].

Interestingly, while Mg^2+^ has often been invoked in the stabilization of RNA motifs for substrate recognition, examples have been reported where RNA aptamers are perfectly capable of recognizing a natural substrate by complexation, while non-native ligands do require metal ions to promote stability [9]. In these cases the metal cofactor presumably makes up for stabilizing contacts that are not formed by the non-native ligand.

### 4.6. Caveats

As previously noted, structural studies often utilize divalent magnesium at concentrations (10–20 mM) that far exceed that of free and available cellular Mg^2+^ (~0.5 mM) [21]. As such, it is not possible to immediately conclude that all identified magnesium binding sites are populated under normal physiological conditions. The work reported herein does, however, provide thermodynamic insight on a selection of the highest affinity sites, and therefore those that are most likely to be of natural functional relevance. 

It is important to note that while the averaged representation of multiple crystallographically defined RNA structures often depict clusters of multiple Mg^2+^ ions, the vast majority of these sites, and particularly the more weakly bound sites, will not be populated at physiological Mg^2+^ concentrations, are not functionally relevant, and will not coexist with proximal neighboring sites as a result of electrostatic repulsion. In fact, most sites in RNA structures are populated in sub-stochiometric limits and do not exist adjacent to each other in a specific RNA molecule.

Finally, in an interesting and relevant report [3], it has been described how the presence of amino acids can influence the ability of divalent ions to bind to RNA. In fact, amino-acid-complexed Mg^2+^ has been demonstrated to enhance RNA function by orders of magnitude over that found with Mg^2+^ alone. Such reports emphasize the fact that studies are often conducted under conditions that do not accurately reflect the cellular environment, and that other metabolites can play a key role in mediating and modulating the cellular chemistry of metal ions.

### 4.7. Conclusions

In conclusion, this work demonstrates the importance of recognizing the role of a thorough evaluation of the thermodynamics of divalent magnesium binding, coupled with studies of molecular dynamics [25,26], to complement the extensive and increasing body of structural information available for metal–RNA contacts. This leads to a more complete understanding of the role of metals, and in particular, divalent magnesium, in controlling the structural and functional activities of RNA [13,14]. Underlying the most critical magnesium binding motifs is the 10-membered-ring moiety that forms the basis for many other recognized Mg^2+^ binding domains, such as the magnesium-clamp and Y-clamp motifs. The higher-affinity 10-membered-ring magnesium binding motif induces a sharp turn in an RNA strand and is further stabilized by a neighboring phosphate, or one further along the chain, to form the required functional structure. High-energy binding offsets the cost of stabilizing the steric constraints and electrostatic repulsions of such a motif, which typically underlies and prioritizes a key functional role.

Outer-sphere coordination to Mg^2+^-bound water molecules is less likely to be used to stabilize a structural feature (which requires higher affinity binding to provide the energy to sustain such a motif), but rather is generally involved in sites that promote electrostatic stabilization. By contrast, the inner-sphere coordination mode emphasizes greater structural definition of tight turns and overcoming electrostatic repulsion between contiguous phosphate groups.

It is significant that the affinity of functionally essential roles appears to be tuned to the physiologically available Mg^2+^ concentrations of 0.2–0.5 mM, while general electrostatic stabilization is most likely assigned to monovalent ions (and in particular, Na^+^), with the higher-charge-density Mg^2+^ ion being utilized where there is an unusual pocket of negative charge density,

Importantly, several caveats must be closely regarded from inspection of crystallographically defined Mg^2+^ binding sites. Principally, many studies show Mg^2+^ to crosslink discrete RNA strands from distinct chains in a crystallographically defined unit cell. While stabilized in an environment defined by crystal packing forces, such sites are unlikely to survive in aqueous solution and no functional role should be inferred from such coordination sites. Moreover, many sites have been identified from structures obtained in the presence of high, and certainly non-physiological, magnesium ion concentrations (usually ~10 to 20 mM).

Finally, it can be stated that the wealth of structural information that continues to be accumulated in RNA biology and biochemistry is providing substantive insights into the roles of metal cofactors in function. This work aims to provide guidance on how to interpret this information as it relates to the role of an often essential divalent magnesium ion. A specific transition point emerges when ∆G ~ −10 kcal/mol, where the functional role can be accommodated by a coordination mode that encompasses both minimal inner-sphere and compensatory outer-sphere modes, while at the lower end of the binding spectrum, with ∆G > −9 kcal mole^−1^, outer-sphere charge neutralization of pockets of negative charge is the norm. Beyond that, simple monovalent ions (Na^+^, and K^+^) are sufficient for charge neutralization of regular single- and double-strand nucleic acid secondary structures, given their higher available cellular or physiological concentrations.

## Figures and Tables

**Table 1:** Experimental values for electrostatic and hydrogen-bonding binding free energies (∆G_b_) of divalent magnesium to RNA [18].

∆G_b_ (kcal mol^−1^) Inner Sphere	∆G_b_ (kcal mol^−1^) Outer Sphere
−3.3	−1.2

**Table 2:** Summary of the three structurally characterized Mg^2+^ binding motifs (Figure 2) with highest binding energies estimated from experimental binding data in the Supplementary Materials file (Table S1). All represent inner-sphere binding motifs, where Pi corresponds to phosphate oxygen. Cis/trans/10-membered-ring coordination motifs are illustrated in Figure 1.

Motif Name	Type
Magnesium clamp	2 × cis-Pi or 2 × trans-Pi
10-membered ring	2 or 3 consecutive Pi in cis-, fac-, or mer- geometries (for 3-coordination the third Pi may be separated by one residue from the 10-membered ring),
	or 4 Pi from two distinct 10-membered-ring pairs
Y-clamp	3 Pi in a mer- geometry where the third Pi is distant to a pair of Pi representing the 10-membered-ring coordination

**Table 3:** Selected sites taken from the Supplementary Materials file (Table S1) as illustrative examples of magnesium binding modes (Figure 3, Figure 4 and Figure 5). Abbreviations include: IS, inner sphere; OS, outer sphere; Pi, backbone phosphate.

pdb id	IS Pi	IS Ribose	IS Other	OS Pi	OS Ribose	OS Base	Binding Free Energy (IS) kcal/mole	Binding Free Energy (OS) kcal/mole	Binding Free Energy (Total) kcal/mole
4enc	A6, U7, U41, G42		F^−^				−16.5	0	−16.5
3oxm	A33, C66	G32				U67, C68, U69	−9.9	−3.6	−13.5
1hr2	A171			C165, U168, C170	U167	U167, A171	−3.3	−7.2	−10.5

## Data Availability

No new data were created or analyzed in this study. Data sharing is not applicable to this article.

## Supplementary Materials

The following supporting information can be downloaded at: https://www.mdpi.com/article/10.3390/life14060765/s1, Table S1: A summary of Mg(II) binding sites described in the manuscript; Figure S1: A visual presentation of the binding data summarized in Table S1.
